# Supplementary material for: Evolution of chemosensory tissues and cells across ecologically diverse Drosophilids
Source: Nat Commun. 2024 Feb 5;15:1047. doi: 10.1038/s41467-023-44558-4 (PMC10844241; doi:10.1038/s41467-023-44558-4)
Supplement: Supplementary file 1 — Supplementary Information [file 41467_2023_44558_MOESM1_ESM.docx]

**Supplementary information**

**Evolution of chemosensory tissues and cells across ecologically diverse *Drosophilids***

Gwénaëlle Bontonou^1,2,*✉,^, Bastien Saint-Leandre^1,2,*,✉^, Tane Kafle^1,2^, Tess Baticle^1^, Afrah Hassan^1^, Juan Antonio Sánchez-Alcañiz^3^, J. Roman Arguello^1,2,4✉^

^1^Department of Ecology & Evolution, Faculty of Biology and Medicine, University of Lausanne, Lausanne, Switzerland

^2^Swiss Institute of Bioinformatics, Lausanne, Switzerland

^3^Instituto de Neurociencias, UMH & CSIC, San Juan de Alicante, Spain

^4^Present address: School of Biological and Behavioural Sciences, Queen Mary University of London, London, UK

*These authors contributed equally

^✉^Corresponding authors: [gwenaelle.bontonou@unil.ch](mailto:gwenaelle.bontonou@unil.ch), [bastien.saint-leandre@unil.ch](mailto:bastien.saint-leandre@unil.ch), [roman.arguello@unil.ch](mailto:roman.arguello@unil.ch)

**Supplementary Figures 1 – 21**

**Supplementary references**


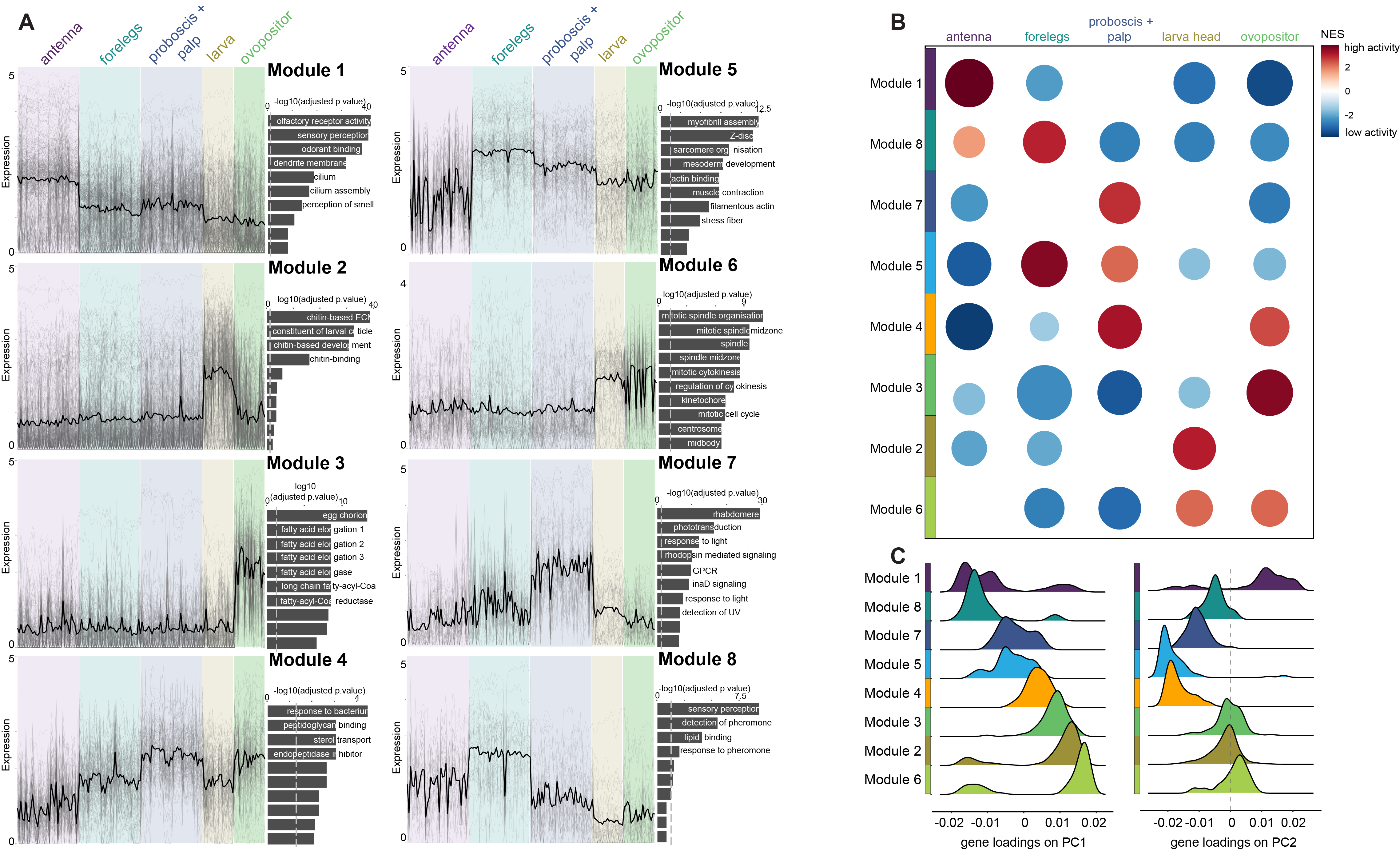


**Supplementary Fig. 1. Identification of expression modules across chemosensory tissues.**

We used CEMITool to identify co-expressed gene modules. From the gene expression table, CEMITool uses an unsupervised filtering method to select the genes used in the analyses. It then uses soft-clustering methods to determine a similarity criterion between pairs of genes. Based on this criterion, genes are separated into modules using the Dynamic Tree Cut package.

**(A)** Gene co-expression analyses showing the expression profiles of individual genes (thin lines) across samples grouped by tissue (left plots). The thick line displays the median expression of all co-expressing genes within a gene module. Right to the profile plots are histograms of enriched pathways ranked by *p*-values. Dashed lines show the significance thresholds.

**(B)** Set Enrichment Analyses displaying the modules’ (from panel A) activity per tissue.

**(C)** Density plots showing the distribution of genes belonging to a module (from panel A) on the first (left) and second (right) principal components of the PCA from Fig. 1B.

Location of source data for this figure can be found in “Source_data.xlsx”.

**
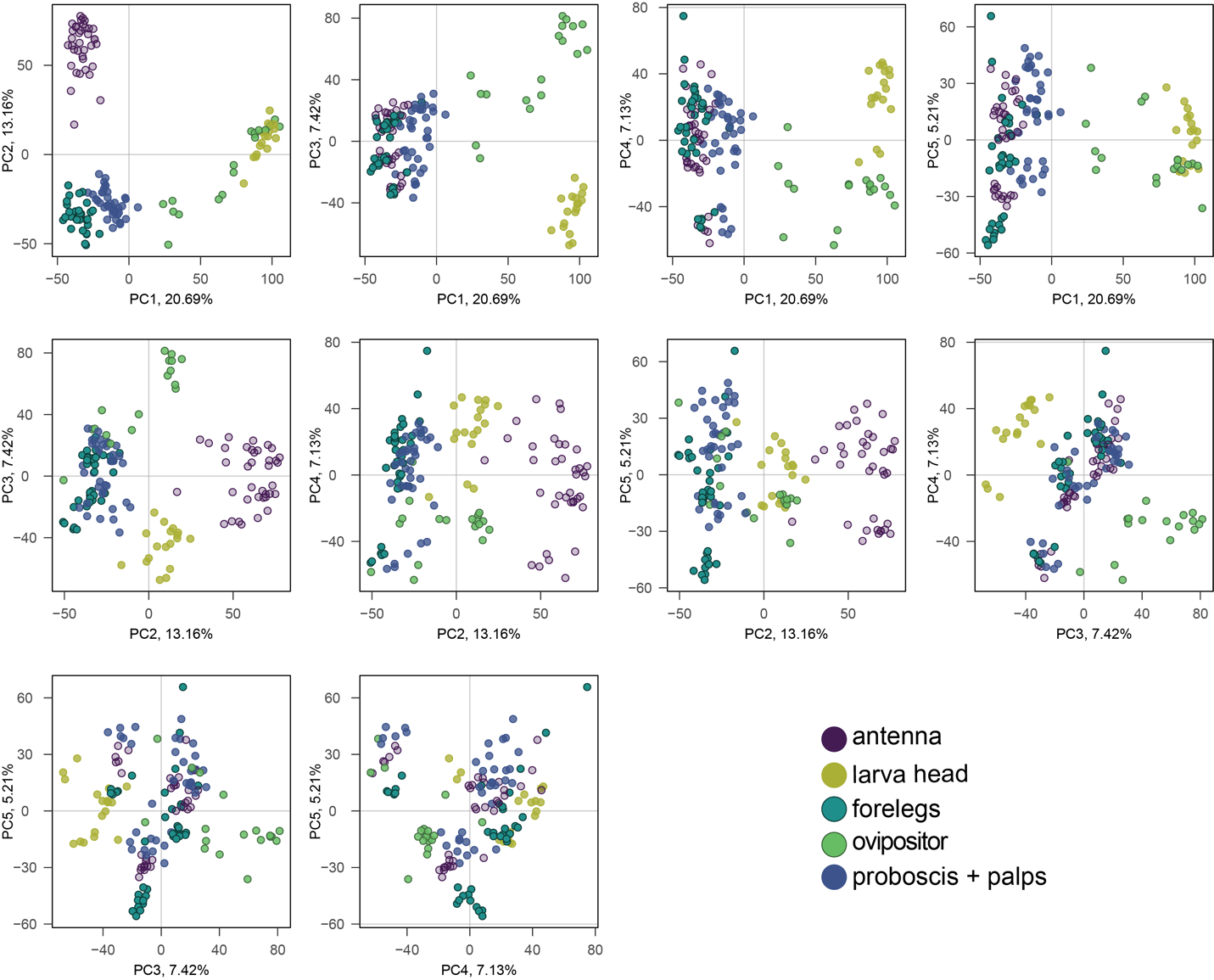
**

**Supplementary Fig. 2. PCA analysis of chemosensory transcriptomes beyond PC 1 and PC 2.**

Across the different principal component pairings, the only two tissues that do not separate are the foreleg and proboscis+palps. Location of source data for this figure can be found in “Source_data.xlsx”.

**
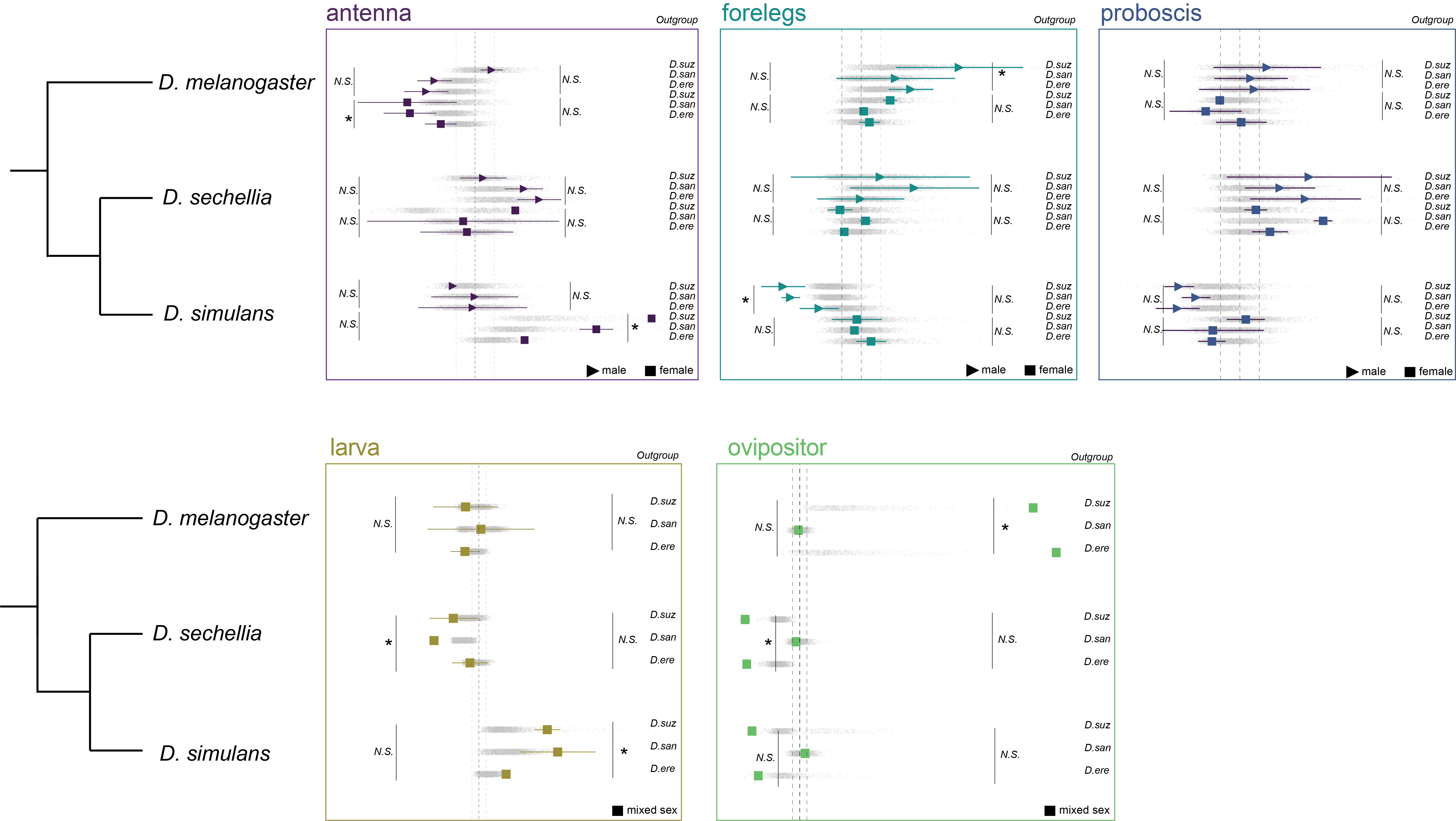
**

**Supplementary Fig. 3. Relative rate tests using different outgroup species.**

Relative rate test results arranged by the species’ phylogeny and tissues. We computed relative rate tests between *D. melanogaster, D. simulans* and *D. sechellia* using either *D. erecta*, *D. santomea*, or *D. suzukii* as the outgroup species as indicated in the right margins of each plot. Colored shapes and lines display the mean and standard deviation of Z-scores from the full set of 1:1 orthologs. Gray data points are Z-scores that resulted from repeating the tests with subsampled datasets (Methods). Asterisks denote the significantly elevated (positive values) or reduced (negative values) rates of gene expression change from all permutations (Wilcoxon test comparing Z-score distribution to the minimum and maximum values of non-significant Z-scores: dotted lines). Outgroup species names are abbreviated to the first three letters. Location of source data for this figure can be found in “Source_data.xlsx”.

**
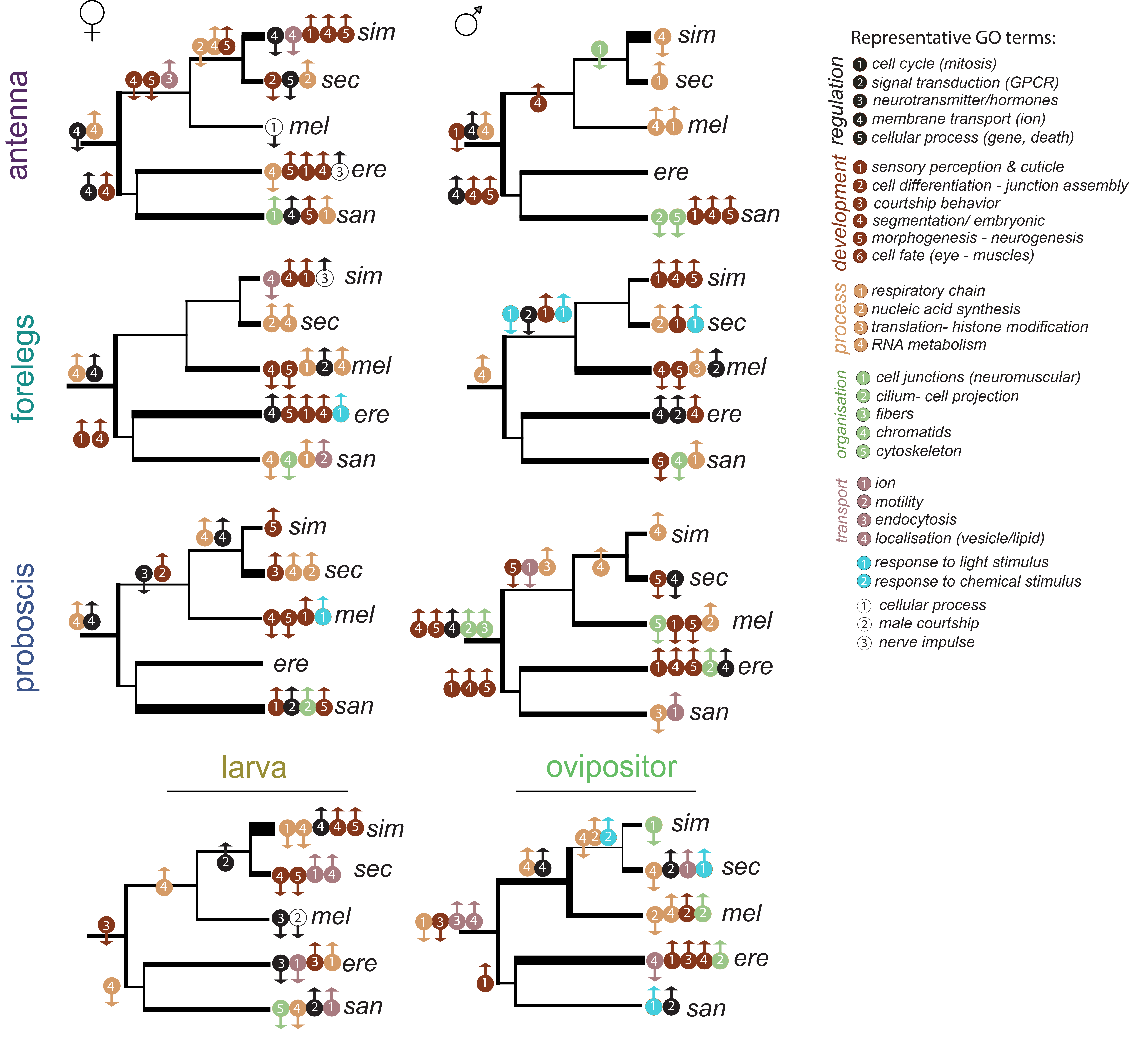
**

**Supplementary Fig. 4. Functional enrichment analysis of differentially expressed genes.**

Gene Ontology (GO) term enrichment analyses of differentially expressed genes for each branch of the species tree and each tissue. Circles correspond to representative terms listed on the right panel. Circles are colored according to large semantic categories and numbers correspond to semantic sub-clusters within these categories. We performed GO term analyses for up-regulated (up arrows) and down-regulated (down arrows) genes separately. Location of source data for this figure can be found in “Source_data.xlsx”.

**
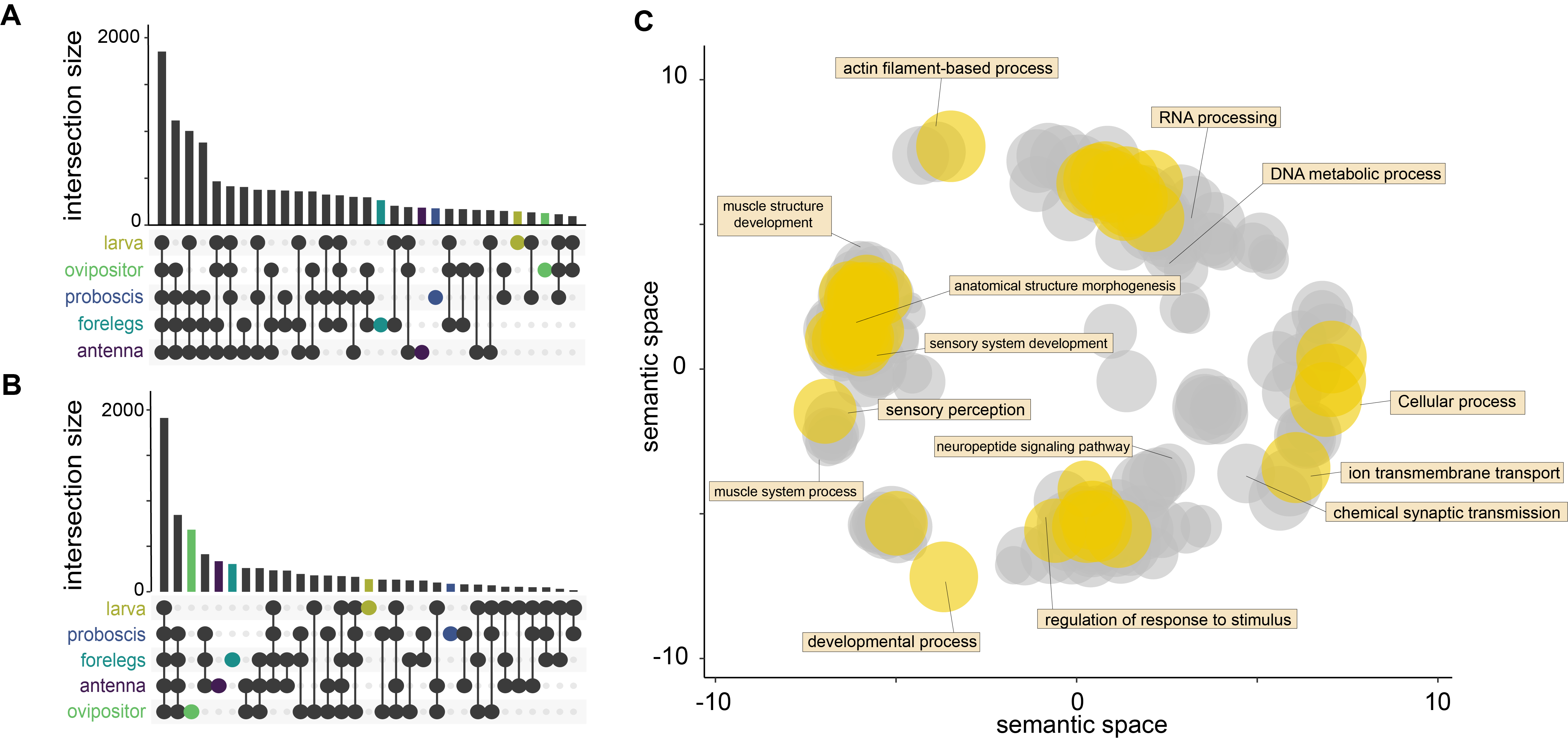
**

**Supplementary Fig. 5. Comparison of the sets of differentially expressed genes identified by the phylogenetically-informed (OU) and DESeq2 methods.**

**(A)** Number of differentially expressed (DE) genes that overlap across tissues (regardless of species) using the l1ou method. Note that this is the same plot as in Fig. 2B and shows that most DE genes are shared across tissues.

**(B)** Number of DE genes that overlap across tissues (regardless of species) using DESeq2 in pair-wise comparisons. DESeq2 gives comparable results to the l1ou method regarding the number of DE genes that are shared across tissues.

**(C)** Multidimensional Scaling (MDS) from the two methods’ GO terms similarity matrix. Semantically similar GO terms project close to each other. Yellow circles show semantic terms shared between the two methods and gray circles show semantic terms unique to one or the other method. GO terms from the two methods clustered together indicating that DE genes from the two methods belong to similarly enriched pathways.

Location of source data for this figure can be found in “Source_data.xlsx”.

**
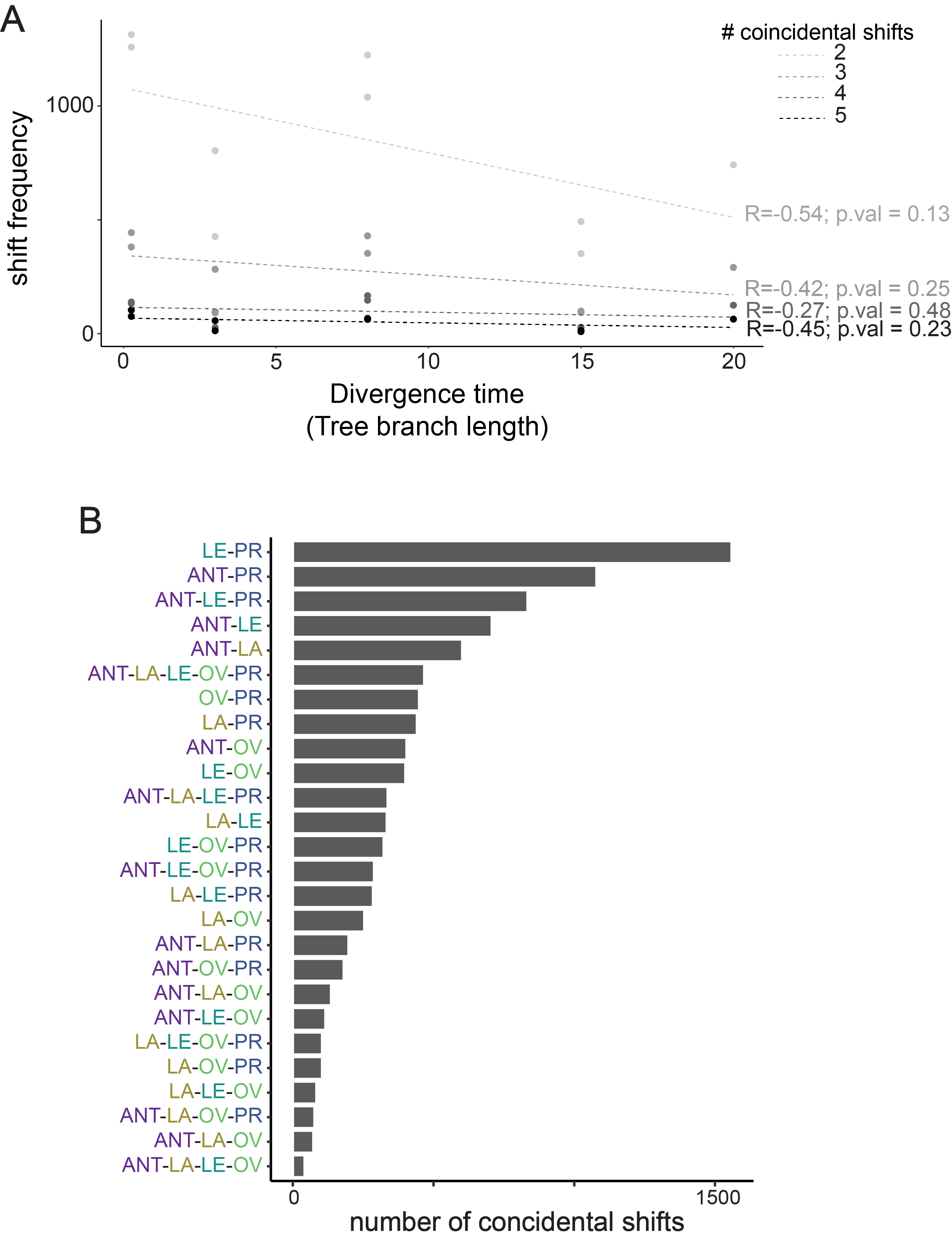
**

**Supplementary Fig. 6. Investigation of coincidental shifts.**

**(A)** Number of coincidental shifts grouped by the number of coincidental changes and according to branch length (i.e. divergence time). The number of coincidental shifts is not correlated with branch length.

**(B)** Frequency of coincidental shifts for all tissue combinations. Overall, the most frequent coincidental shift occurred between the legs and proboscis+palps.

Location of source data for this figure can be found in “Source_data.xlsx”.

**
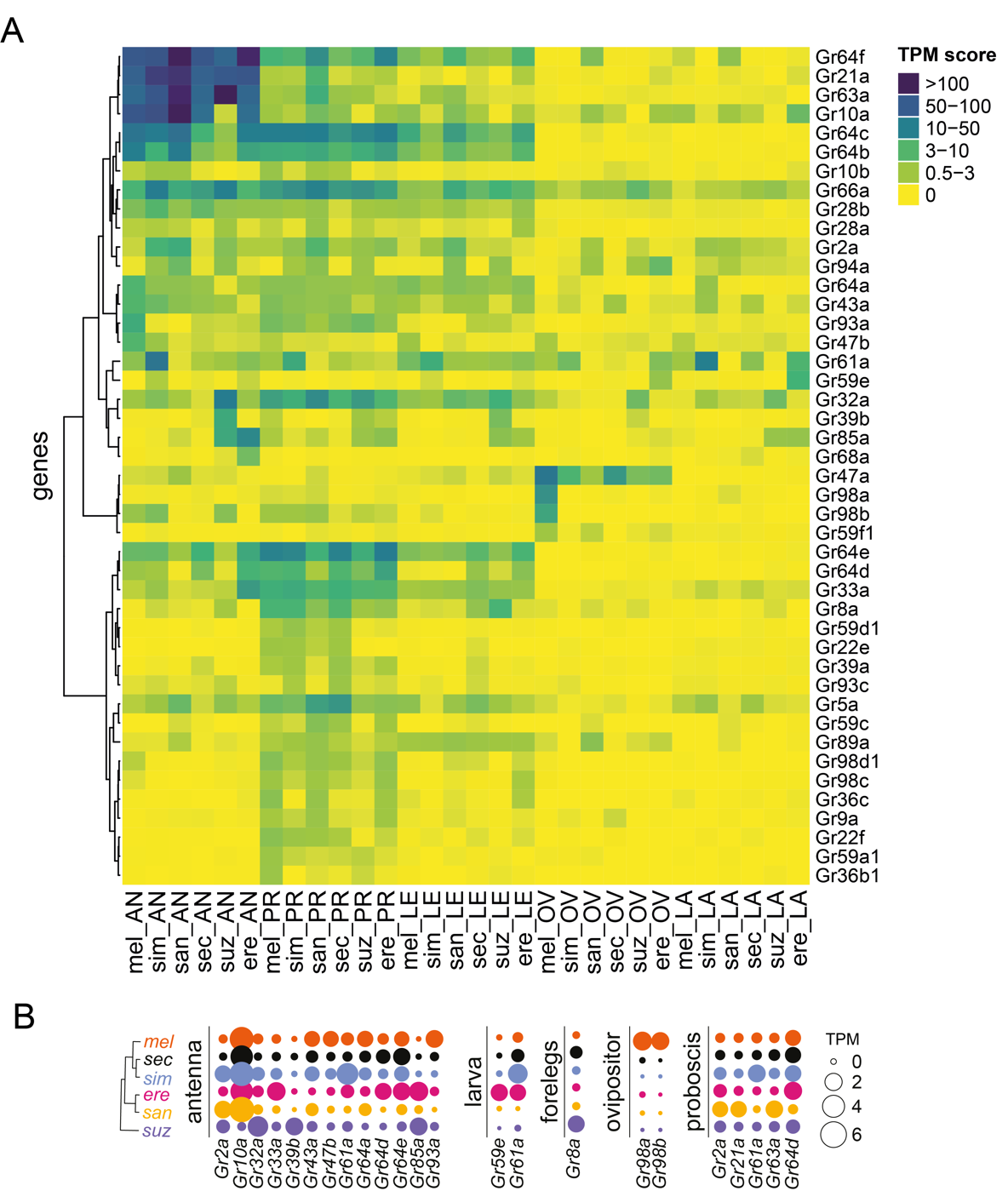
**

**Supplementary Fig. 7. *Grs* expression across species and tissues.**

**(A)** Hierarchical clustering of mean *Gr* expression values (TPM). Each row contains a gene and each column contains a species’ tissue sample. Clustering was performed gene-wise. AN=antenna, PR=proboscis, LE=forelegs, OV= ovipositor, LA=larval head.

**(B)** *Gr*s that have evolved species-specific expression gains or losses. Species names are abbreviated to the first three letters.

Location of source data for this figure can be found in “Source_data.xlsx”.

**
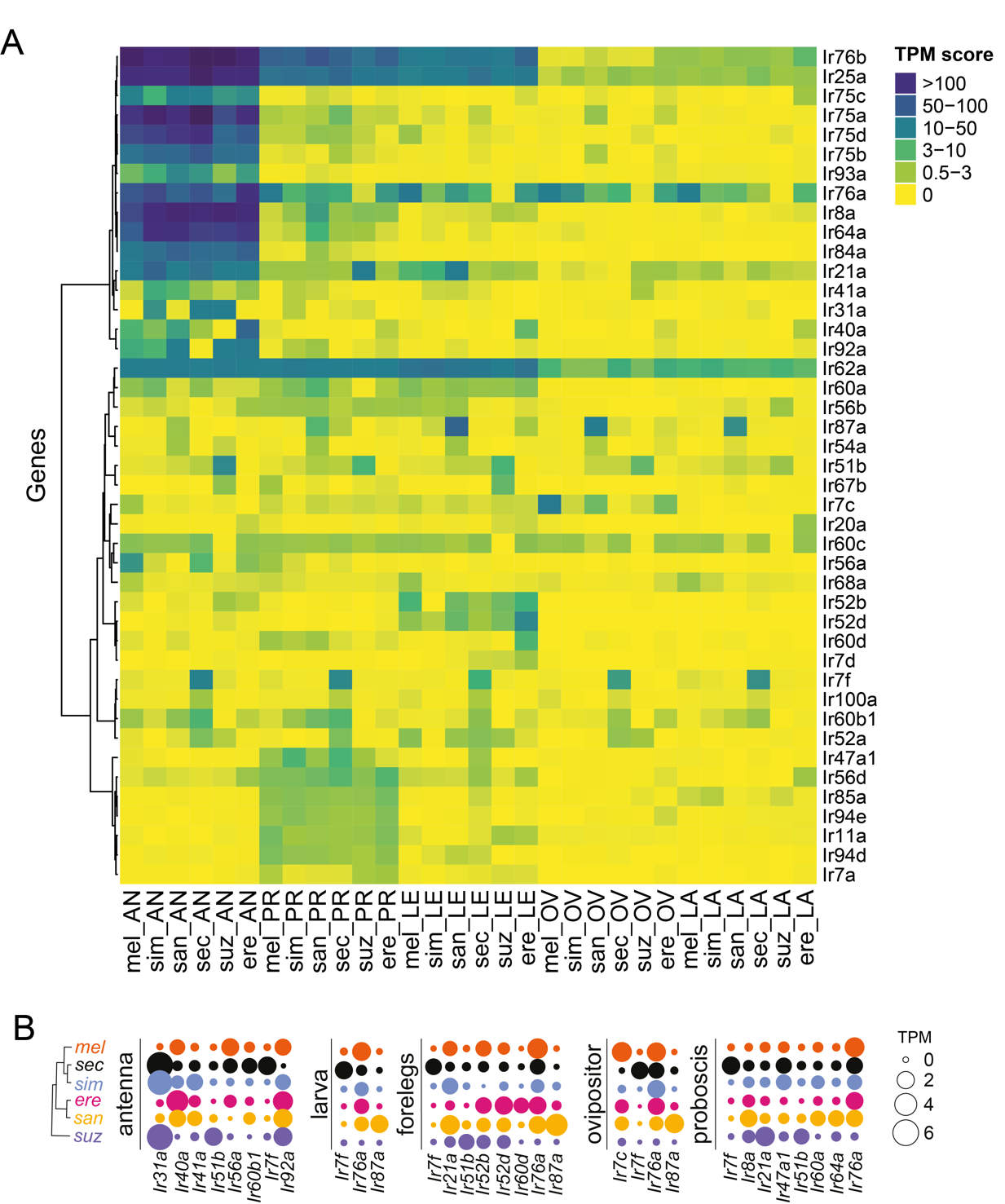
**

**Supplementary Fig. 8. *Irs* expression across species and tissues.**

**(A)** Hierarchical clustering of mean *Ir* expression values (TPM). Each row represents a gene and each column a sample. Clustering was performed gene-wise. AN=antenna, PR=proboscis, LE=forelegs, OV= ovipositor, LA=larval head.

**(B)** *Ir*s that have evolved species-specific expression gains or losses. Species names are abbreviated to the first three letters.

Location of source data for this figure can be found in “Source_data.xlsx”.

**
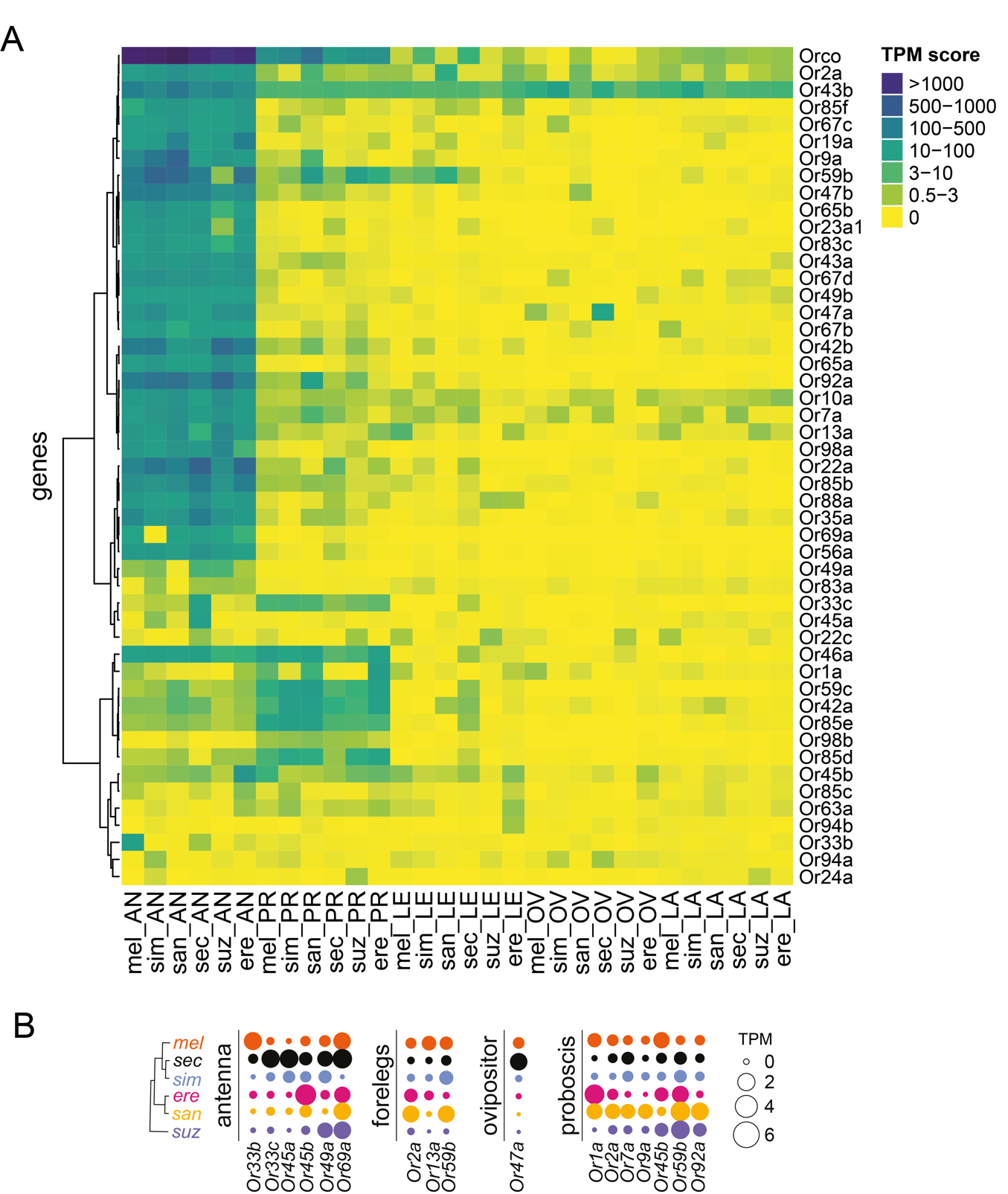
**

**Supplementary Fig. 9. *Ors* expression across species and tissues.**

**(A)** Hierarchical clustering of mean *Ors* expression values (TPM). Each row represents a gene and each column a sample. Clustering was performed gene-wise. AN=antenna, PR=proboscis, LE=forelegs, OV= ovipositor, LA=larval head.

**(B)** *Ors* that have evolved species-specific expression gains or losses. Species names are abbreviated to the first three letters.

Location of source data for this figure can be found in “Source_data.xlsx”.

**
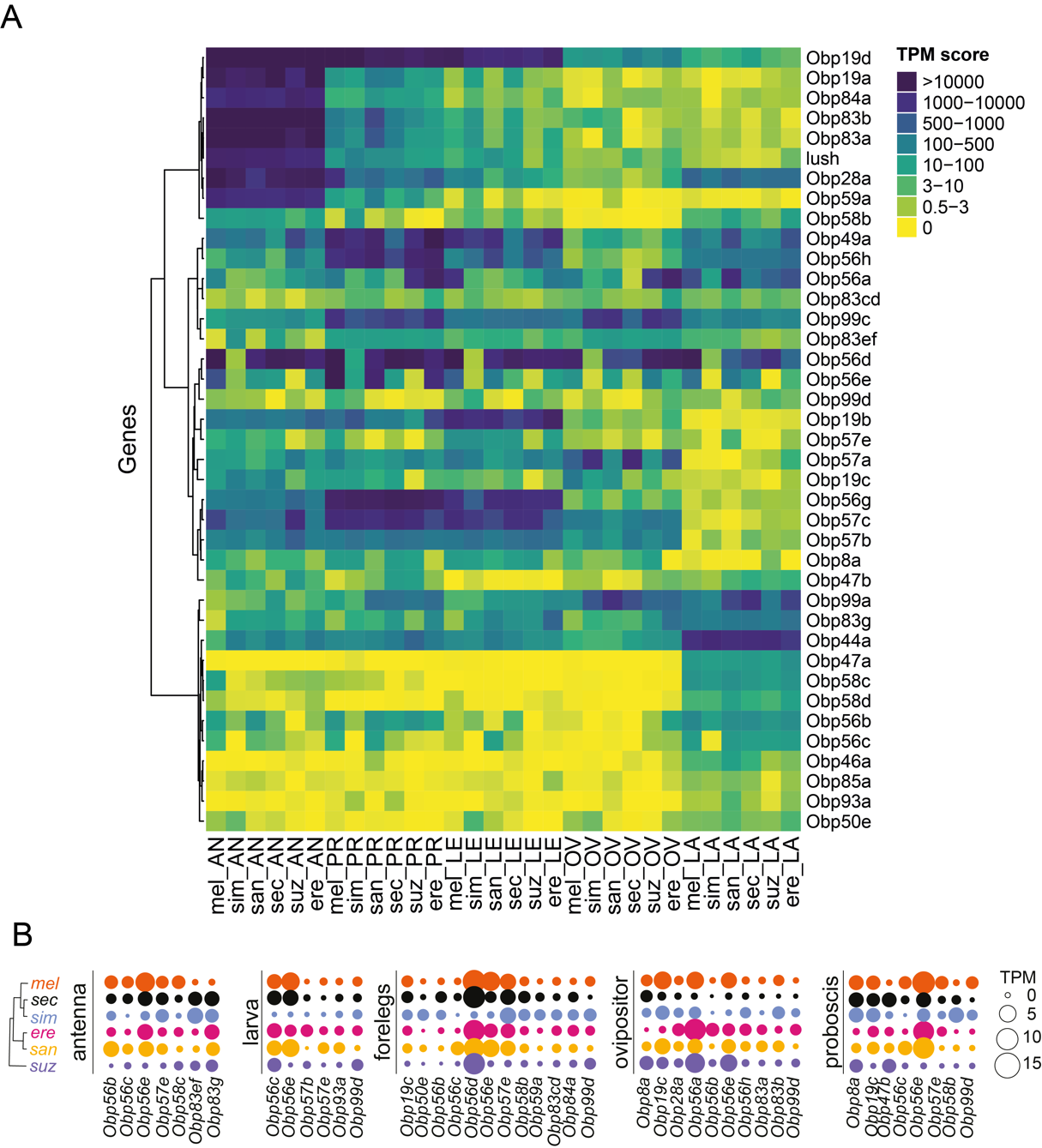
**

**Supplementary Fig. 10. *Obps* expression across species and tissues.**

**(A)** Hierarchical clustering of mean *Obps* expression values (TPM). Each row represents a gene and each column a sample. Clustering was performed gene-wise. AN=antenna, PR=proboscis, LE=forelegs, OV= ovipositor, LA=larval head.

**(B)** *Obp*s that have evolved species-specific expression gains or losses. Species names are abbreviated to the first three letters.

Location of source data for this figure can be found in “Source_data.xlsx”.

**
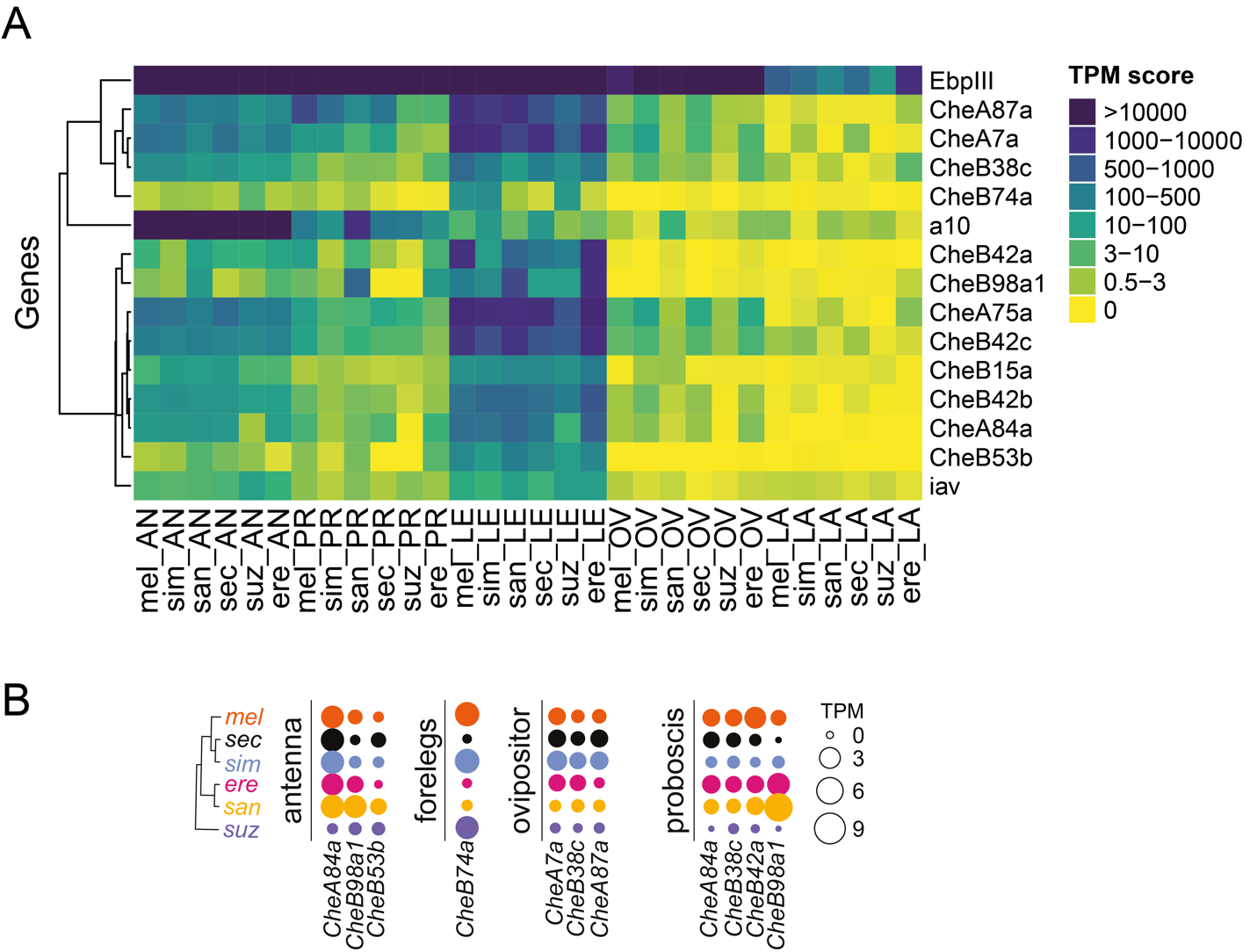
**

**Supplementary Fig. 11. *CSPs* expression across species and tissues.**

**(A)** Hierarchical clustering of mean *CSP* expression values (TPM). Each row represents a gene and each column a sample. Clustering was performed gene-wise. AN=antenna, PR=proboscis, LE=forelegs, OV= ovipositor, LA=larval head.

**(B)** *CSP*s that have evolved species-specific expression gains or losses. Species names are abbreviated to the first three letters.

Location of source data for this figure can be found in “Source_data.xlsx”.

**
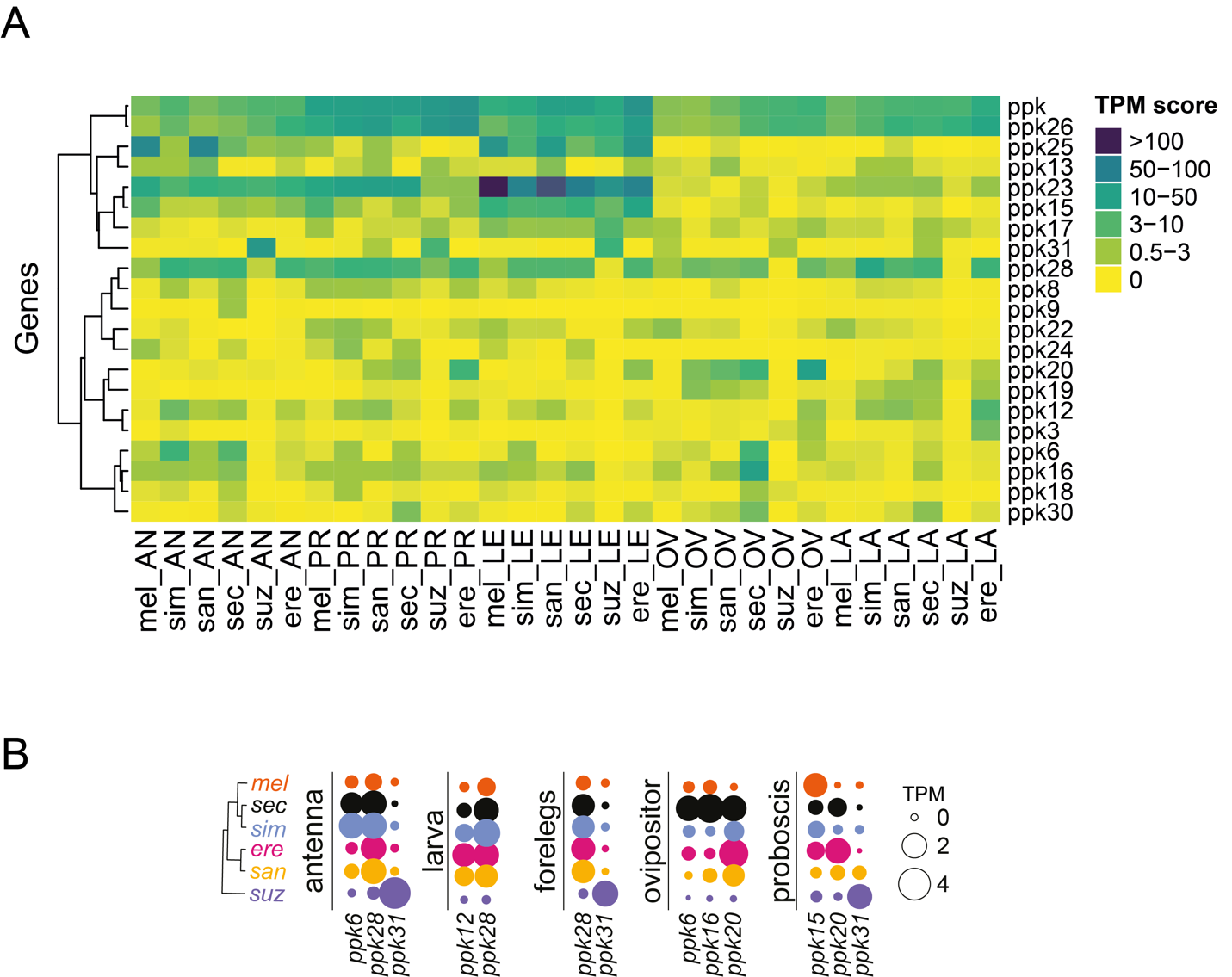
**

**Supplementary Fig. 12. *Ppks* expression across species and tissues.**

**(A)** Hierarchical clustering of mean *ppks* expression values (TPM). Each row represents a gene and each column a sample. Clustering was performed gene-wise. AN=antenna, PR=proboscis, LE=forelegs, OV= ovipositor, LA=larval head.

**(B)** *ppk*s that have evolved species-specific expression gains or losses. Species names are abbreviated to the first three letters.

Location of source data for this figure can be found in “Source_data.xlsx”.

**
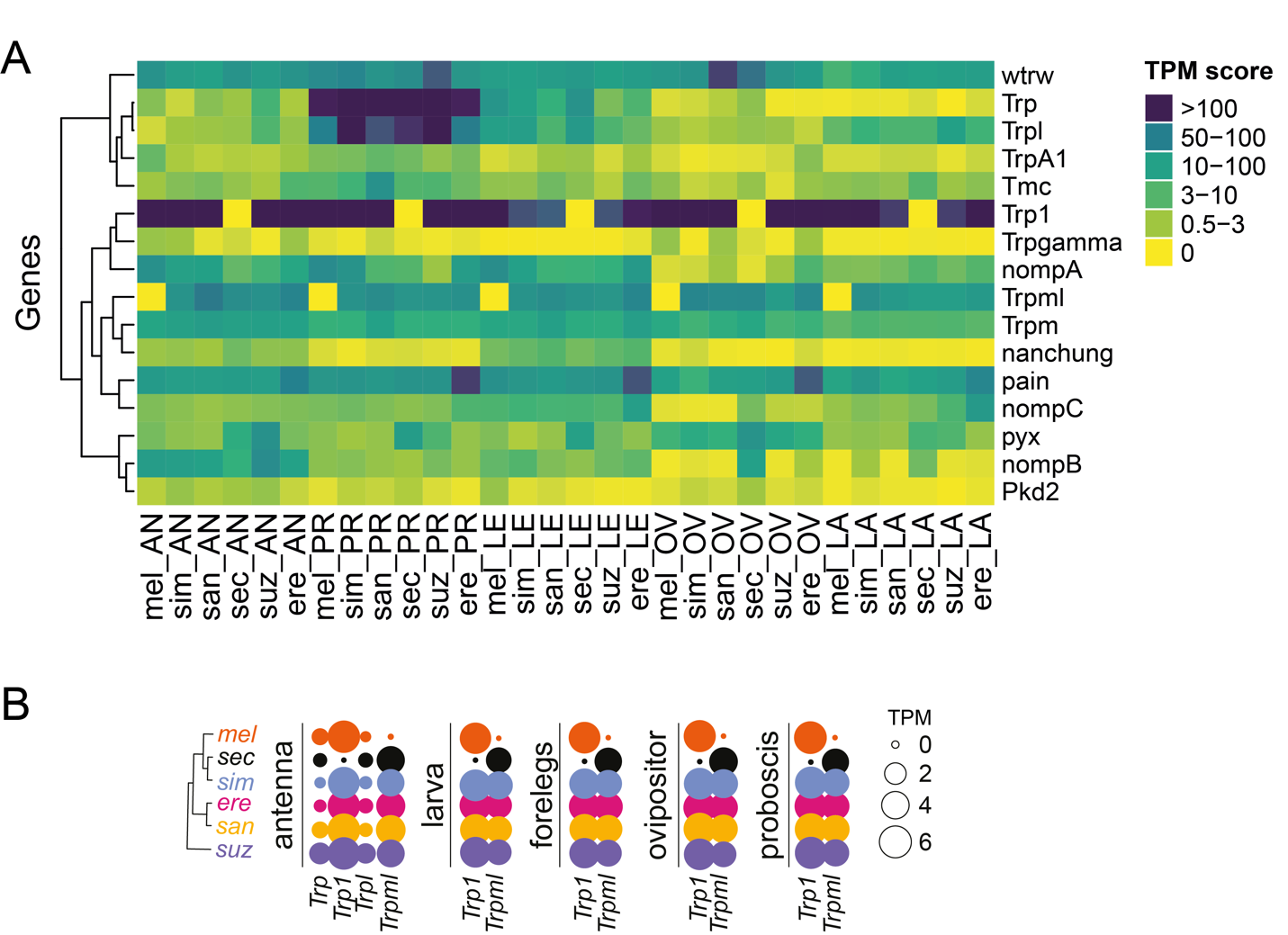
**

**Supplementary Fig. 13. *Trps* expression across species and tissues.**

**(A)** Hierarchical clustering of mean *Trp* expression values (TPM). Each row represents a gene and each column a sample. Clustering was performed gene-wise. AN=antenna, PR=proboscis, LE=forelegs, OV= ovipositor, LA=larval head.

**(B)** *Trp*s that have evolved species-specific expression gains or losses. Species names are abbreviated to the first three letters.

Location of source data for this figure can be found in “Source_data.xlsx”.





**Supplementary Fig. 14. Expression of duplicated *Gr,* *Ir* and *Or* family members across tissues and species.**

Mean expression values (TPM) of *Gr*, *Ir* and *Or* paralogs in *D. erecta, D. santomea, D. sechellia, D. simulans* and *D. suzukii* with the exclusion of the *Gr59a* and *Ir52* subfamilies (which are displayed in Fig S16; no recent duplicates were identified in *D. melanogaster*). Each row contains a gene and each column contains a species’ tissue sample. AN=antenna, PR=proboscis, LE=forelegs, OV= ovipositor, LA=larval head. Species names are abbreviated to the first three letters. Location of source data for this figure can be found in “Source_data.xlsx”.





**Supplementary Fig. 15. Expression of duplicated *CSPs*, *Obps*, *Trps* and *ppks* family members across tissues and species.**

**(A)** Mean expression values (TPM) of *CSPs* and *Obps* paralogs in *D. erecta, D. santomea, D. simulans* and *D. suzukii* (no recent duplicates were identified in *D. melanogaster*).

**(B)** Mean expression values (TPM) of *Trps* and *ppks* paralogs *D. santomea, D. sechellia, D. simulans* and *D. suzukii*. Each row contains a gene and each column contains a species’ tissue sample. AN=antenna, PR=proboscis, LE=forelegs, OV= ovipositor, LA=larval head. Species names are abbreviated to the first three letters. Location of source data for this figure can be found in “Source_data.xlsx”.


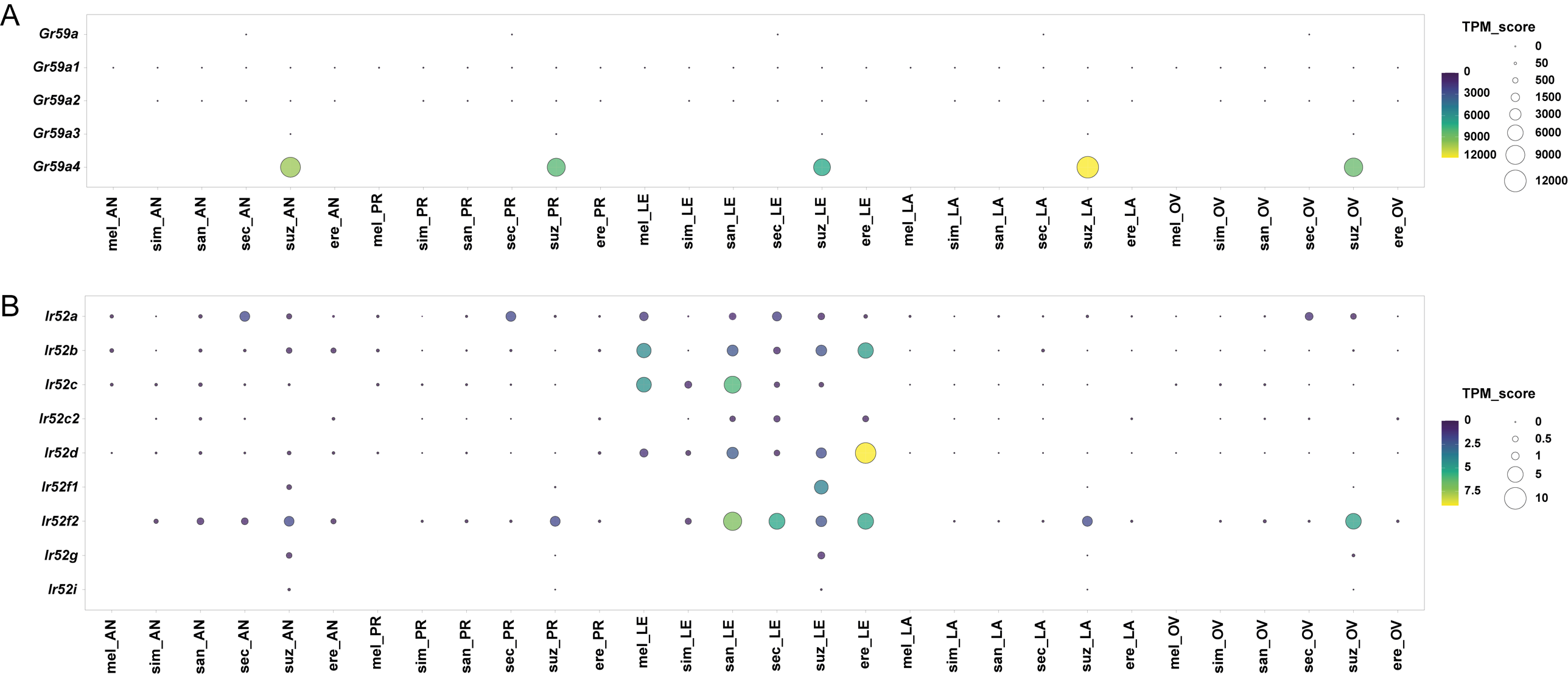


**Supplementary Fig. 16. Duplicated chemosensory gene subfamilies whose members have evolved expression gains.**

**(A)** Mean expression values (TPM) of the *Gr59a* paralogs across species and tissues.

**(B)** Mean expression values (TPM) the *Ir52* clade members across species and tissues. Each row contains a gene and each column contains a species’ tissue sample. AN=antenna, PR=proboscis, LE=forelegs, OV= ovipositor, LA=larval head. Species names are abbreviated to the first three letters. Location of source data for this figure can be found in “Source_data.xlsx”.

**
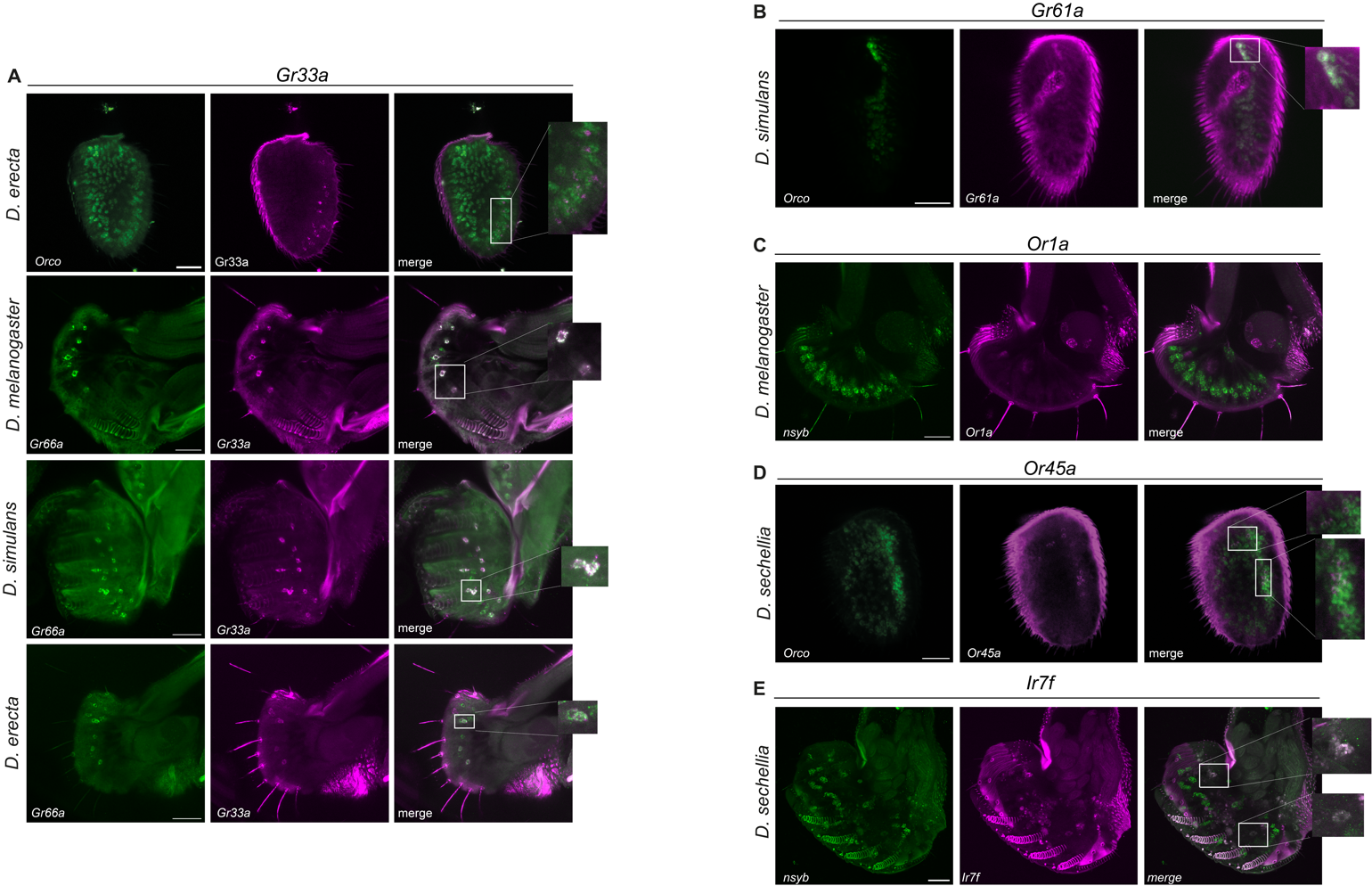
**

**Supplementary Fig. 17. Analyses of chemosensory genes with lineage-specific gains of expression.**

**(A)** *Gr33a* antenna-specific gain of expression in *D. erecta*. Upper row: *in situ* Hybridization chain reaction (HCR) experiment using *Gr33a* and *Orco* probes in a whole mount *D. erecta* antenna. Co-labelled cells are magnified in the right panels. Rows 2-4: *in situ* HCR using *Gr66a* and *Gr33a* probes in the labellum of *D. melanogaster*, *D. simulans* and *D. erecta*. Co-labelled cells are magnified in the right panels. We observed conserved RNA-seq expression of *Gr33a* in the legs and proboscis samples across the six species but also found a high level of expression in *D. erecta*’s antenna. Consistent with our RNA-seq data, we were able to detect the expression of *Gr33a* in *D. erecta* antenna (17±4 cells) but not in the other two species. The *D. erecta*-specific antennal signal was verified by the successful detection of *Gr33a* in the proboscis of *D. simulans*, *D. sechellia*, and *D. melanogaster*, where *Gr33a* and *Gr66a* are co-expressed in bitter sensing neurons as previously described^1^. A co-labeling experiment using an *Orco* probe, which labels all olfactory sensory neurons, revealed *Gr33a*-*Orco* co-expression in the antenna, indicating that *D. erecta*’s *Gr33a* has gained olfactory sensory neuron expression while maintaining its role in bitter taste sensing.

**(B)** *in situ* HCR using *Orco* and *Gr61a* probes in *D. simulans* antenna. Co-labelled cells are magnified in the right panels. *Gr61a* has been identified as a glucose receptor in *D. melanogaster* and is expressed in neurons within the labellum, tarsal leg segments, and the labral sense organ^2-4^. Consistent with these descriptions, we have detected expression of *Gr61a* in these same tissues in *D. simulans* and *D. melanogaster* (mean TPM respectively in the labellum: 7.4 and 0.60, in the front legs: 5.3 and 2.13), in the legs of *D. santomea*, *D. sechellia*, *D.erecta* and *D. suzukii* (mean TPM = 0.67, 0.62, 1.39 and 0.82, respectively) and additionally in the antenna of *D. simulans*, *D. melanogaster*, *D. suzukii* and *D. erecta* (mean TPM = 23, 1.25, 0.53 and 1.49, respectively). A *D. simulans*-specific *in situ* probe confirmed the antennal olfactory sensory neurons expression of *Gr61a*. Though the signal for this probe was weak, we estimated ~6 *Gr61a*-expressing cells.

**(C)** *in situ* HCR using *Or1a* and *nsyb* probes in the labellum of *D. melanogaster*. *Or1a* has been described as larva-specific in *D. melanogaster* and involved in attraction to some odors^5^. Within our RNA-seq dataset, we found a high level of expression in the proboscis of *D. erecta* (mean TPM = 24.05) and appreciable levels of expression in the proboscis of *D. melanogaster and* *D. santomea* (mean TPM = 2.78 and 5.72, respectively). Consistent with these results, *in situ* HCR using an *Or1a* probe localized its expression in the proboscis of these three species but not in *D. simulans* (see Fig. 4D). Interestingly, the *Or1a* probe labeled cells larger than typical chemosensory neurons and located in a region that has not been described to contain sensory cells. To test if these cells belong to an unexpected population of neurons, we carried out a co-labelling experiment in *D. melanogaster* using the *Or1a* probe and a probe for a pan-neuronal marker, *neuronal Synaptobrevin* (*nSyb*). We detected broad *nSyb* expression throughout the proboscis but no co-localization with the *Or1a* probe. This result indicates that *Or1a* has evolved a non-neuronal expression in *D. erecta, D. santomea* and *D. melanogaster*. Based on their cuboidal morphology, we hypothesized that cells expressing *Or1a* are part of the salivary tract.

**(D)** *in situ* HCR using *Orco* and *Or45a* probes in a whole mount antenna of *D. sechellia*. Co-labelled cells are magnified in the right panels. The expression of *Or45a* has been described as larva-specific in *D. melanogaster* and involved in aversive behavior to some odors^6-7^. While we did not detect *Or45a* in our *D. melanogaster* larval head RNA-seq datasets, likely due to low expression and/or the small number of cells that express it (mean TPM = 0.05), we did observe a high level of expression specifically in *D. sechellia* antenna (mean TPM = 20.25). Using HCR probes for *Or45a*, we were able to detect the expression *in D. sechellia*’s antennal olfactory sensory neurons (11±4 cells) but not in *D. simulans* or *D. melanogaster* antennae.

**(E)** *in situ* HCR using *nSyb* and *Ir7f* probes in the proboscis of *D. sechellia*. Co-labelled cells are magnified in the right panels. Ir7f belongs to a cluster of 7 tandemly arrayed paralogous Ionotropic receptors^8^. This cluster of Irs have diverse expression patterns in adults and larva, with *Ir7f* expression described as being specific to the dorsal pharyngeal sense organ in *D. melanogaster* larva^9^. Recently, a member of this clade, Ir7a, was shown to be an acetic acid sensor^10^, suggesting that the other Ir7 paralogs may recognize additional acids, though no functional data exist for them. In our RNA-seq experiments, we detected only low levels of expression for *Ir7f* in the larva of the six species (TPM<0.3) but found a lineage-specific gain of *Ir7f* expression for the other tissues in *D. sechellia*. We co-labelled *Ir7f* and *nSyb* in *D. sechellia* labial palps, indicating its neuronal expression. Together our expression quantifications and cell detection suggest that *D. sechellia Ir7f* may play a specific function for this species which is an extreme host specialist that resides on noni fruit^11^.

**
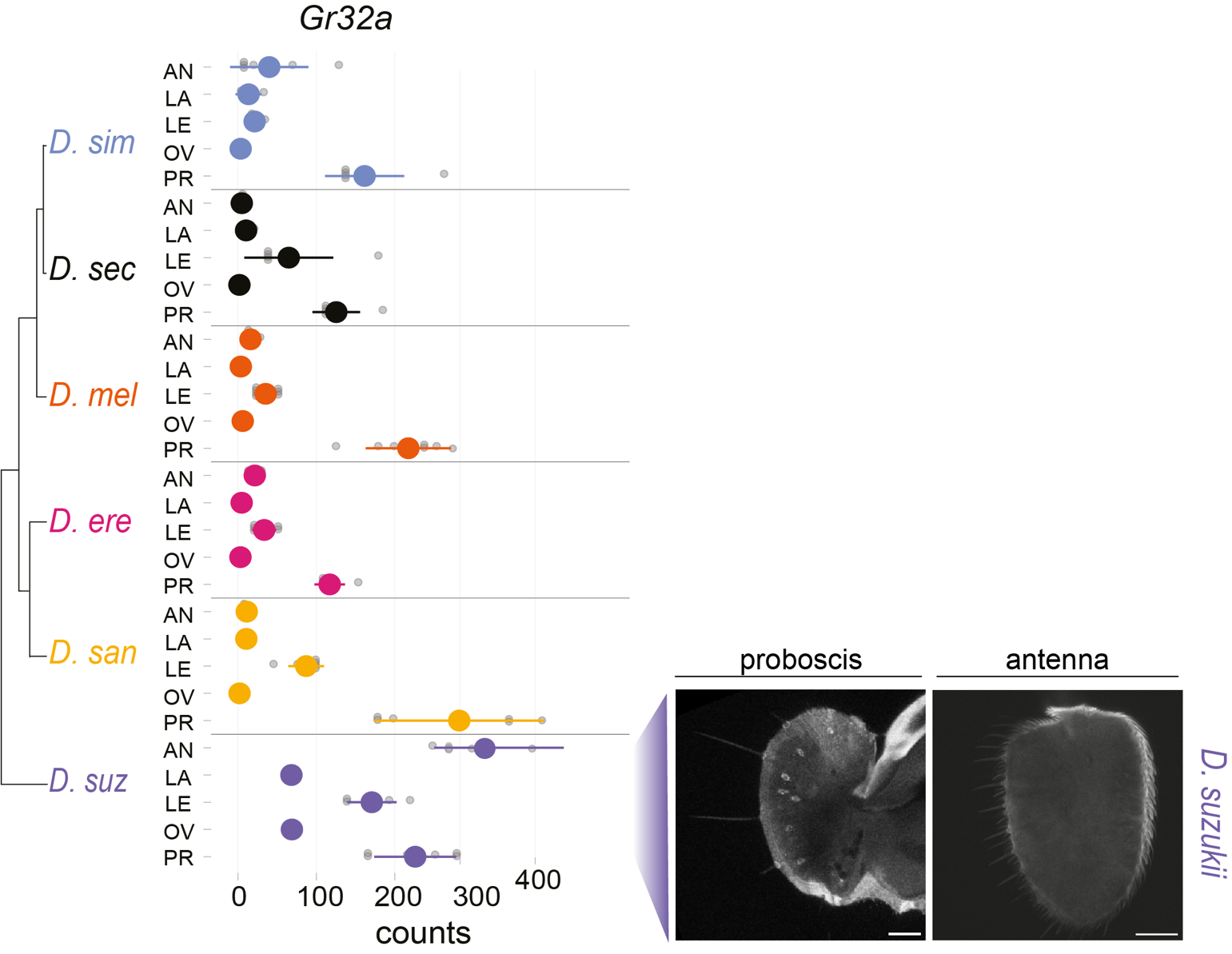
**

**Supplementary Fig. 18. Expression of *Gr32a*.**

RNA-seq expression analyses indicated that *Gr32a* is expressed in all five of *D. suzukii*’s tissues that we examined (left half of figure). Small grey dots are the normalized counts for each replicate and the large colored dots with lines display the mean and standard deviation for a given dataset. To the right are results from an *in situ* HCR experiment using a *D. suzukii*-specific *Gr32a* probe carried out on proboscis and antenna. *Gr32a*-expressing cells were detected in the labellum, indicating a functional probe. For unknown reasons we were unable to detect expression of *Gr32a* in the antenna.

**
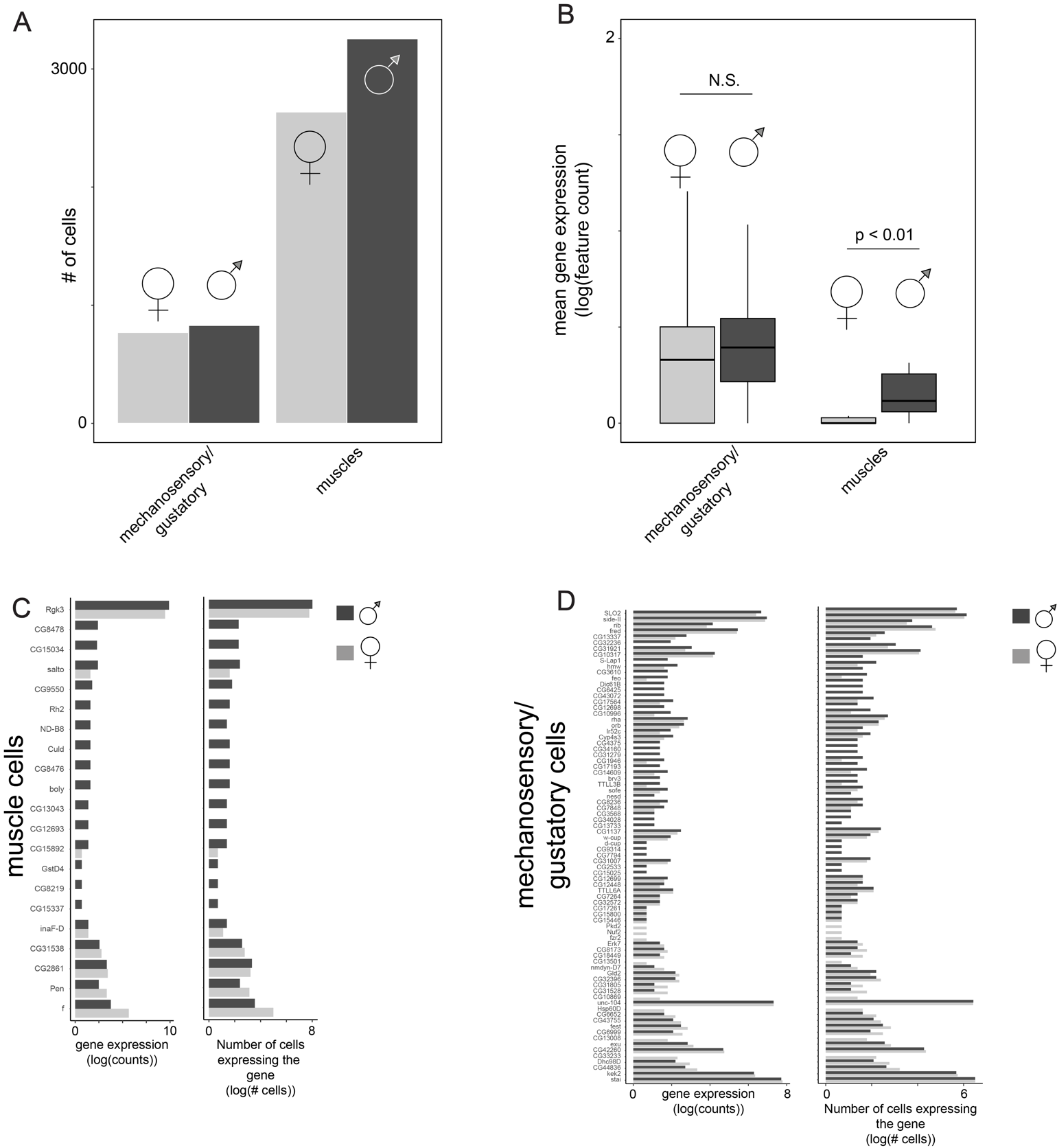
**

**Supplementary Fig. 19. Comparison of male and female leg atlases.**

(A) Number of female and male cells in cell clusters enriched in male-biased genes from the Fly Cell Atlas dataset. (B) Boxplot of mean expression for male-biased genes in female (light gray) and male (dark gray) cells. Significance from a Wilcoxon test between male and female mean expression are displayed. (C, D) Bar plots displaying each individual gene’s contribution to the pooled expression and cell numbers shown in panels A and B. Location of source data for this figure can be found in “Source_data.xlsx”.

**
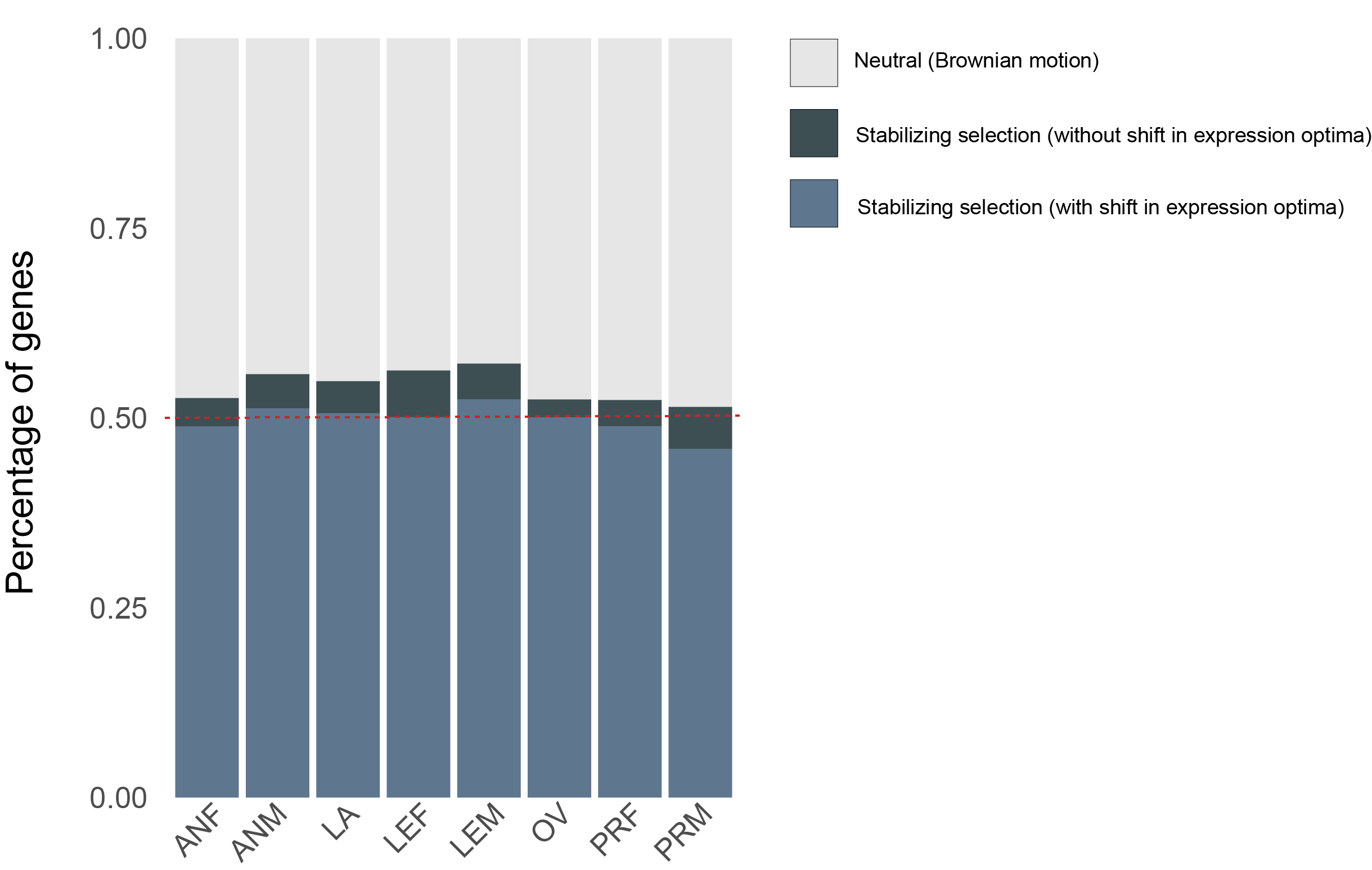
**

**Supplementary Fig. 20. Models best fitting expression changes.**

Percentage of genes found to best fit one of three evolutionary models (for all 1:1 orthologs across all tissues): neutrally evolving, constrained, or constrained with a divergent branch(s). Location of source data for this figure can be found in “Source_data.xlsx”.

**
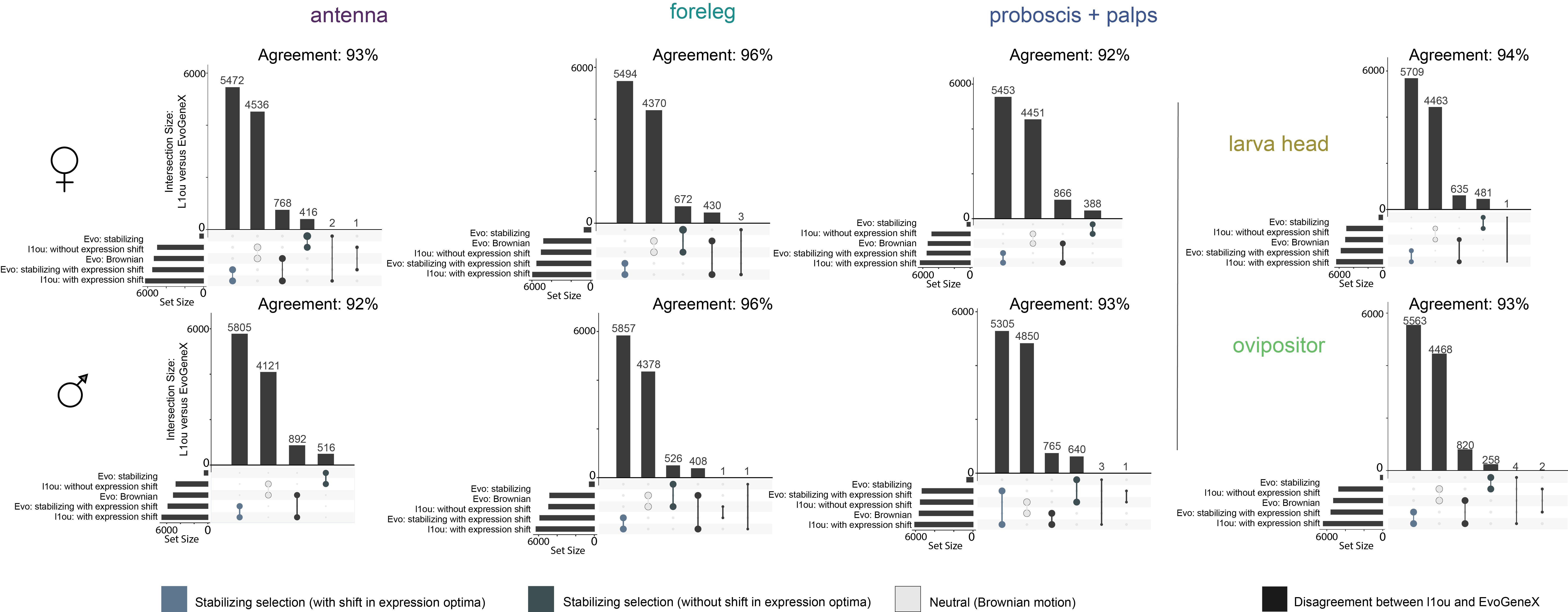
**

**Supplementary Fig. 21. Gene expression divergence agreement between l1ou and EvoGeneX.**

Number of genes that overlap across two statistical methods for detecting gene expression divergence. Most differentially expressed genes detected by the l1ou method are best explained by an O.U. model with multiple expression optima. Most genes found to be non-differentially expressed by the l1ou method overlap with genes best fitting a Brownian motion model (neutral evolution) or an O.U. model with only one expression optimum (stabilizing selection).

Location of source data for this figure can be found in “Source_data.xlsx”.

**Supplementary references**

1. Moon, S. J., Lee, Y., Jiao, Y. & Montell, C. A Drosophila gustatory receptor essential for aversive taste and inhibiting male-to-male courtship. *Curr Biol* **19**, 1623–7 (2009).

2 Kohatsu, S., Tanabe, N., Yamamoto, D. & Isono, K. Which Sugar to Take and How Much to Take? Two Distinct Decisions Mediated by Separate Sensory Channels. *Front. Mol. Neurosci.* **15**, (2022).

3. Dahanukar, A., Lei, Y.-T., Kwon, J. Y. & Carlson, J. R. Two Gr Genes Underlie Sugar Reception in Drosophila. *Neuron* **56**, 503–516 (2007).

4. Miyamoto, T., Chen, Y., Slone, J. & Amrein, H. Identification of a Drosophila Glucose Receptor Using Ca2+ Imaging of Single Chemosensory Neurons. *PLOS ONE***8**, e56304 (2013).

5. Fishilevich, E. *et al.* Chemotaxis behavior mediated by single larval olfactory neurons in Drosophila. *Curr Biol* **15**, 2086–96 (2005).

6. Kreher, S. A., Kwon, J. Y. & Carlson, J. R. The Molecular Basis of Odor Coding in the Drosophila Larva. *Neuron* **46**, 445–456 (2005).

7. Bellmann, D. *et al.* Optogenetically induced olfactory stimulation in Drosophila larvae reveales the neuronal basis of odor-aversion behavior. *Front. Behav. Neurosci.* **4**, (2010).

8. Croset, V. *et al.* Ancient Protostome Origin of Chemosensory Ionotropic Glutamate Receptors and the Evolution of Insect Taste and Olfaction. *PLoS Genet.* **6**, e1001064 (2010).

9. Sánchez-Alcañiz, J. A. *et al.* An expression atlas of variant ionotropic glutamate receptors identifies a molecular basis of carbonation sensing. *Nat. Commun.* **9**, 4252 (2018).

10. Rimal, S. *et al.* Mechanism of Acetic Acid Gustatory Repulsion in Drosophila. *Cell Rep.* **26**, 1432-1442.e4 (2019).

11. R’Kha, S., Capy, P. & David, J. R. Host-plant specialization in the Drosophila melanogaster species complex: a physiological, behavioral, and genetical analysis. *Proc. Natl. Acad. Sci.* **88**, 1835–1839 (1991). *Neuron* **46**, 445–456 (2005).
