# Supplementary material for: Evolution of chemosensory tissues and cells across ecologically diverse Drosophilids
Source: Nat Commun. 2024 Feb 5;15:1047. doi: 10.1038/s41467-023-44558-4 (PMC10844241; doi:10.1038/s41467-023-44558-4)
Supplement: Supplementary file 3 — Description of Additional Supplementary Files [file 41467_2023_44558_MOESM3_ESM.pdf]

Description of Supplementary Data files:

Supplementary Data 1: Differentially expressed genes based on all 1:1 orthologs. This table contains the branch and tissue in which the expression changes occurred.

Supplementary Data 2: Gene Ontology (GO) terms associated with differentially expressed genes. This table contains GO terms enriched for differentially expressed genes (up- and down-regulated) on each tree branch for all tissues. The table also includes the list of differentially expressed genes contributing to significant GO term enrichment.

Supplementary Data 3: The list of chemosensory genes analyzed and the gene families that they belong to.

Supplementary Data 4: Orthologous Chemosensory genes expression results compared with previous descriptions in the literature. Genes with a TPM > 0.5 were defined as expressed. Each sheet is dedicated to a tissue. The "Summary" sheet presents the summary for all tissue comparisons. The "References" sheet provides the full reference list used to carry out the comparisons.

Colored cells indicate the following:
green: 1:1 ortholog that has species-specific expression.
blue: expressed genes in our datasets but not in the literature.
yellow: agreement with tissue expression but different species (possible polymorphism)
red: no match
purple: species-specific paralog

Values within cells indicate the following:
"ND": denotes genes for which we could not find any evidence of expression in the literature.
"N": denotes a gene absent in a given species.

Supplementary Data 5: The list of genes expressed in an individual tissue across all species.

Supplementary Data 6: Differentially expressed genes based on the curated chemosensory gene sets of 1:1 orthologs. This table contains the branch where the shift occurred and the tissues involved.

Supplementary Data 7: TPM values of each chemosensory 1:1 orthologs (first sheet) and mean expression values of duplicated chemoreceptors (second sheet).

Supplementary Data 8: Sex-biased genes separated by species and tissues. The *p*-values are based on a Wald test. The significance thresholds for these analyses were a log(fold change) > 1.5 and an (FDR) adjusted $p < 0.01$.

Supplementary Data 9: Detailed information on the molecular reagents used in this project.

Supplementary Data 10: The GTFs outputted by BRAKER.

Supplementary Data 11: Lookup table connecting Flybase gene IDs to the gene symbols outputted by BRAKER for all 1:1 orthologs.

Supplementary Data 12: RNA-seq Count tables for the entire dataset generated by HTseq.

Supplementary Data 13: TPM count tables based on File S2 for all 1:1 orthologs.

Supplementary Data 14: GTF files based on conserved genic regions between the six species' ("trimmed" GTFs) for the set of 1:1 orthologs.

Supplementary Data 15: RNA-seq Count tables based on the "trimmed" GTF file (File S4) generated by HTseq.

Supplementary Data 16: Lookup table connecting Flybase gene IDs to the gene symbols outputted by BRAKER for all genes (1:1 orthologs and lineage-specific duplicates).

Supplementary Data 17: GTF files for the manually curated set of chemosensory genes.

Supplementary Data 18: TPM count table for the manually annotated chemosensory gene set.
