# Supplementary material for: Evolution of chemosensory tissues and cells across ecologically diverse Drosophilids
Source: Nat Commun. 2024 Feb 5;15:1047. doi: 10.1038/s41467-023-44558-4 (PMC10844241; doi:10.1038/s41467-023-44558-4)
Supplement: Supplementary file 22 — Reporting Summary [file 41467_2023_44558_MOESM22_ESM.pdf]

nature portfolio

Corresponding author(s): Gwénaëlle Bontonou, Bastien Bastien Saint-Leandre, J. Roman Arguello

Last updated by author(s): 2023/12/07

Reporting Summary

Nature Portfolio wishes to improve the reproducibility of the work that we publish. This form provides structure for consistency and transparency in reporting. For further information on Nature Portfolio policies, see our Editorial Policies and the Editorial Policy Checklist.

Statistics

For all statistical analyses, confirm that the following items are present in the figure legend, table legend, main text, or Methods section.

n/a	Confirmed	
☐	☒	The exact sample size (*n*) for each experimental group/condition, given as a discrete number and unit of measurement
☒	☐	A statement on whether measurements were taken from distinct samples or whether the same sample was measured repeatedly
☐	☒	The statistical test(s) used AND whether they are one- or two-sided<br>*Only common tests should be described solely by name; describe more complex techniques in the Methods section.*
☐	☒	A description of all covariates tested
☐	☒	A description of any assumptions or corrections, such as tests of normality and adjustment for multiple comparisons
☐	☒	A full description of the statistical parameters including central tendency (e.g. means) or other basic estimates (e.g. regression coefficient) AND variation (e.g. standard deviation) or associated estimates of uncertainty (e.g. confidence intervals)
☐	☒	For null hypothesis testing, the test statistic (e.g. *F*, *t*, *r*) with confidence intervals, effect sizes, degrees of freedom and *P* value noted<br>*Give P values as exact values whenever suitable.*
☒	☐	For Bayesian analysis, information on the choice of priors and Markov chain Monte Carlo settings
☒	☐	For hierarchical and complex designs, identification of the appropriate level for tests and full reporting of outcomes
☐	☒	Estimates of effect sizes (e.g. Cohen's *d*, Pearson's *r*), indicating how they were calculated

Our web collection on statistics for biologists contains articles on many of the points above.

Software and code

Policy information about availability of computer code

Data collection	All software packages (and their versions) used to collect the data in this study are listed in the Methods. Our own code and implementation of existing software is available on our gitlab page: https://gitlab.com/EvoNeuro/sensory-rnaseq.<br>Antenna, legs and proboscis data from the FlyCell Atlas were from: https://flycellatlas.org/
Data analysis	All software packages (and their versions) used to analyze the data in this study are listed in the Methods. Our own code and implementation of existing software is available on our gitlab page: https://gitlab.com/EvoNeuro/sensory-rnaseq.<br>In a nutshell, software used for:<br>-gene annotations: BRAKER v2.1.6, Augustus v3.4.0, BUSCO v3.0.2, Cufflinks v2.2.1<br>-orthogroups annotations and analysis: OrthoFinder v2.3.8, EMBOSS v6.6.0 , Possvm v1.1, MAFFT v7.490, IQ-TREE v2.2.0.5, BLAST v2.10.1<br>-RNA-seq data analysis STAR v2.7.8, HTseq v0.11.2,DESeq2 v1.34.0, MAFFT v7.475<br>-Transcriptomic clustering and Relative rate tests: TreeExp v0.99.3, R v4.1.2<br>-Differential expression analyses: l1ou R package v1.43, EvoGeneX v0.9.9.0, CEMITools v1.18.1, DESeq2 v1.34.0, R v4.1.2<br>- Manual curation of chemosensory gene set: Geneious v2022.0.2, Minimap2 v2.17<br>- Fly Cell atlas data manipulation: R package Seurat v4.3.0, SeuratObject v4.1.3, SeuratDisk v0.0.0.9020, R v4.1.2

For manuscripts utilizing custom algorithms or software that are central to the research but not yet described in published literature, software must be made available to editors and reviewers. We strongly encourage code deposition in a community repository (e.g. GitHub). See the Nature Portfolio guidelines for submitting code & software for further information.

Data

Policy information about availability of data

All manuscripts must include a data availability statement. This statement should provide the following information, where applicable:

- Accession codes, unique identifiers, or web links for publicly available datasets
- A description of any restrictions on data availability
- For clinical datasets or third party data, please ensure that the statement adheres to our policy

The data generated for this project are available at ArrayExrpress under the accession code E-MTAB-12656 : https://www.ebi.ac.uk/biostudies/arrayexpress/studies/E-MTAB-12656?query=E-MTAB-12656 and on our lab's "sensory RNAseq" GitLab repository: https://gitlab.com/EvoNeuro/sensory-rnaseq. The normalized count data for 1:1 orthologs can be explored and plotted with our CT2 dashboard available at: https://ctct.unil.ch/.

Research involving human participants, their data, or biological material

Policy information about studies with human participants or human data. See also policy information about sex, gender (identity/presentation), and sexual orientation and race, ethnicity and racism.

Reporting on sex and gender	N/A
Reporting on race, ethnicity, or other socially relevant groupings	N/A
Population characteristics	N/A
Recruitment	N/A
Ethics oversight	N/A

Note that full information on the approval of the study protocol must also be provided in the manuscript.

Field-specific reporting

Please select the one below that is the best fit for your research. If you are not sure, read the appropriate sections before making your selection.

☐ Life sciences ☐ Behavioural & social sciences ☒ Ecological, evolutionary & environmental sciences

For a reference copy of the document with all sections, see nature.com/documents/nr-reporting-summary-flat.pdf

Ecological, evolutionary & environmental sciences study design

All studies must disclose on these points even when the disclosure is negative.

Study description	Transcriptomic data from from the main chemosensory organs of closely related Drosophila species (D. melanogaster, D. sechellia, D. simulans, D. santomea, D. erecta, and D. suzukii) from larava (head) and adults (antenna, forelegs, proboscis with maxillary palps, ovipositors (female only) for both males and females. Three or more replicates per sample were collected.
Research sample	Species, source/stock center, and strains used:<br>D. melanogaster, our lab, GDL B54<br>D. simulans, National Drosophila Species Stock Center at Cornell, 14021-0251.008<br>D. sechellia, National Drosophila Species Stock Center at Cornell, 14021-0271.07<br>D. santomea, National Drosophila Species Stock Center at Cornell, 14021-0271.00<br>D. erecta, National Drosophila Species Stock Center at Cornell, 14021-0224.01<br>D. suzukii, Kyorin, K-AWA036<br><br>Tissues used for mRNA library preparation and HCR RNA-FISH experiments were collected from third-instar larvae or non-virgin adults between 2-10 days. For mRNA library preparation the samples collectes were the proboscis and maxillary palps from males and females, the antennas from males and females, the forelegs from males and females, the females ovipositors and the larval heads from mixed sex.
Sampling strategy	Transcriptomic datasets were collected in at least triplicate so that estimates of error could be calculated. HCR RNA-FISH experiments were replicated at least twice.
Data collection	Tissues were collected by manual dissections as described in Methods; Transcriptomic datasets was generated according to the details in Methods; HCR RNA-FISH experiments were collected according to the details in Methods. G.B., J.S.-A. and J.R.A recorded

	data on mRNA library production. A.H., G.B. and T.B. recorded data on HCR experiments. G.B., B.S.-L. and J.R.A recorded data on transcriptomic analyses.
Timing and spatial scale	mRNA libraries data collections started in March 2019 and finished in December 2020. HCR RNA-FISH imaging data was collected between October 2021 and June 2023. The flies were coming from Drosophila stock centers National Drosophila Species Stock at Cornell,and Kyorin (Japan) and were kept inside our lab in Lausanne.
Data exclusions	Some mRNA libraries had been excluded and redone due to QC failing - too few reads or poor mapping rate.
Reproducibility	Several mRNA extractions and mRNA library preparations had to be redone because they did not meet our quality standards (low concentration of bad quality ratios). Once a final working protocol was settled on for the HCR experiments, these experiments were repeated at least twice with the same protocol to ensure their reproducibility (all were reproducible).
Randomization	Tissue dissections, mRNA exctractions and mRNA libraries synthesis were randomized. HCR experiments were performed on multiple days on multiple species and samples: the order of the dissections or the order of samples treatments were randomized.
Blinding	While dissecting, each pool of samples (eg, antenna, labellum, larvae, legs or ovipositor) coming from a given species was given a tracking number written in an excel sheet. RNA extractions were performed in a randomized and blinded way and the amount of recovered RNA was then updated for each sample on the excel sheet. The samples were then randomly taken for the mRNA libraries synthesis but, as each library needed to be coupled with indexes, that step couldn't be done blindly. HCR experiments needed the use of specific probes and amplifiers and thus could not be done blindly.

Did the study involve field work? ☐ Yes ☒ No

Reporting for specific materials, systems and methods

We require information from authors about some types of materials, experimental systems and methods used in many studies. Here, indicate whether each material, system or method listed is relevant to your study. If you are not sure if a list item applies to your research, read the appropriate section before selecting a response.

Materials & experimental systems

n/a	Involved in the study
☒	☐ Antibodies
☒	☐ Eukaryotic cell lines
☒	☐ Palaeontology and archaeology
☐	☒ Animals and other organisms
☒	☐ Clinical data
☒	☐ Dual use research of concern
☒	☐ Plants

Methods

n/a	Involved in the study
☒	☐ ChIP-seq
☒	☐ Flow cytometry
☒	☐ MRI-based neuroimaging

Animals and other research organisms

Policy information about studies involving animals; ARRIVE guidelines recommended for reporting animal research, and Sex and Gender in Research

Laboratory animals	Drosophila lab stocks were used: D. melanogaster B54, D. simulans 14021-0251.008, D. sechellia 14021-0271.07, D. santomea 14021-0271.00, D. erecta 14021-0224.01, D. suzukii K-AWA036.<br>Tissues used for mRNA library preparation and HCR RNA-FISH experiments were collected from third-instar larvae or non-virgin adults between 2-10 days. Please see the Methods section for details on the nature and the amount of tissues collected.
Wild animals	No wild animals were used.
Reporting on sex	Sex was considered in the study and sex-separated data is available.
Field-collected samples	No field-caught samples were used.
Ethics oversight	All experiments were conducted in accordance with ethical guidelines from the University of Lausanne.

Note that full information on the approval of the study protocol must also be provided in the manuscript.

Plants

Seed stocks	N/A
Novel plant genotypes	N/A
Authentication	N/A
